# Perfection should not hinder progress towards occupational exposure limits for psychosocial hazard: A reply to Guseva Canu and van der Molen

**DOI:** 10.5271/sjweh.4313

**Published:** 2026-07-01

**Authors:** Roman Pauli, Jessica Lang, Andreas Müller, Yacine Taibi, Thomas Kraus, Yannick Metzler

**Affiliations:** 1 Institute for Occupational, Social and Environmental Medicine, RWTH Aachen University, Germany.; 2 Department of Work and Organizational Psychology, University of Duisburg-Essen, Germany.; 3 Occupational Health Management, thyssenkrupp Steel Europe AG, Duisburg, Germany.; 4 Leibniz Research Centre for Working Environment and Human Factors, Dortmund, Germany.

We thank Guseva Canu and van der Molen for their thoughtful commentary (1) on our article advocating for the development of occupational exposure limits (OEL) for psychosocial hazards. We appreciate their careful consideration of both the conceptual and methodological challenges associated with transferring established approaches from the assessment of non-psychosocial (ie, chemical and physical) hazards to the psychosocial domain. We also welcome that the authors share the concerns raised in our original discussion paper (2) regarding uncertainties in existing operationalizations of psychosocial hazards. Nonetheless, psychosocial risk assessment has progressed substantially and is considerably more nuanced than suggested in the commentary. While our discussion paper was framed as a mapping of the current knowledge – explicitly titled around “what we know and what we do not know, yet” – we believe the commentary engages primarily with a narrowed reading of our argument, namely a proposal to immediately establish OEL, rather than the roadmap of conceptual and methodological requirements we presented. Considering recent methodological advances, we conclude that advancing toward OEL for psychosocial hazards is not a premature leap but a timely and necessary next step.

## From latent variable models to actionable items

It is true that, for decades, influential work-stress theories have relied on latent variable models to operationalize psychological phenomena which, although not directly observable, have repeatedly been linked to employee health outcomes. Methodologically, manifestations of distinct individual aspects of the latent constructs are combined to operationalize the latent variable of interest. It should be noted, however, that eg, the latent variable *job demands* represents no more an observable hazard than generic categories like *dust* or *metal* represent identifiable hazardous substances. Accordingly, while the goal is to optimize job demands or job control, this can only be accomplished by addressing the distinct actionable items that constitute such latent constructs. Guseva Canu and van der Molen themselves use the example of bus drivers for whom *efforts* had been disentangled into 39 distinct actionable items. As an additional example, the Joint German Occupational Safety and Health Strategy (3) identifies more than 70 actionable items that constitute six latent domains of job stressors (work content, work organization, working time, social relations, work equipment, work environment) each representing a quantifiable presence or absence of hazards. The “fundamental paradigm shift […] toward more specific, measurable, and harmonized approaches”, as advocated by Guseva Canu and van der Molen, is thus already underway. Whether no-observed adverse effect level (NOAEL)/ lowest-observed-adverse effect level (LOAEL) are most usefully defined at the level of individual items, item composites, or aggregated constructs is itself an empirical question that future research should address.

## Subjective bias and social construction of hazards

Guseva Canu and van der Molen argue that assessments of social support, decision authority, or emotional demands are inherently subjective or socially constructed. By referencing cultural norms and economic pressures, they highlight individual processes of perception, implying that actual exposure cannot be accurately quantified in terms of OEL. We believe that these phenomena can still serve as valid and meaningful decision aids for preventive action: Within occupational groups, employees show high within-group agreement in psychosocial risk assessments, and in addition, there is substantial agreement between employee evaluations and those conducted by occupational safety and health (OSH) committees for the same job activities (4). Moreover, methodological advances are improving our understanding of how subjective bias can be minimized. Stress measures frequently confounded exposure with appraisal as items often incorporate evaluations of job characteristics as stressful. To address this, it was recommended to operationalize job stress using condition-related, non-evaluative items (5, 6). Finally, advanced understanding of the psychometrics of psychosocial risk assessment, including activity-based rather than person-based item wordings combined with frequency rather than agreement response options, can further reduce subjective bias via personality traits on assessments (7). These developments demonstrate that, despite inherent subjectivity, psychosocial exposures can be reliably quantified when validated and carefully designed instruments are used.

## Concluding remarks

As with chemical and physical hazards, decisions regarding psychosocial exposures should be guided as objectively as possible by evidence-based criteria, giving both employees and organizations the confidence that preventive measures – or the decision not to implement them – are justified rather than based on subjective judgments. We believe that, despite their construct-dependent ontological nature, OEL for psychosocial hazards can provide substantial support for preventive decision-making. We also share Guseva Canu’s and van der Molen’s emphasis on the political economy of the OEL setting, which our own call for institutionalized discussion forums (Essential 4) explicitly anticipates. With our proposal, we aimed to establish a framework for these decisions based on the current *scientific* state of the art. While its methodological foundations may not be perfect, we believe it represents a substantial improvement to harmonize research efforts and standardize reporting in a way that encourages researchers to tackle the remaining challenges. In other words, act on OEL where the evidence permits and continue to improve where gaps remain.
